# Maturity Prediction in Yellow Peach (*Prunus persica* L.) Cultivars Using a Fluorescence Spectrometer

**DOI:** 10.3390/s20226555

**Published:** 2020-11-17

**Authors:** Alessio Scalisi, Daniele Pelliccia, Mark Glenn O’Connell

**Affiliations:** 1 Agriculture Victoria, Tatura, VIC 3616, Australia; mark.oconnell@agriculture.vic.gov.au; 2 Food Agility CRC Ltd., Ultimo, NSW 2007, Australia; 3 Rubens Technologies Pty Ltd., Rowville, VIC 3178, Australia; 4 Instruments & Data Tools Pty Ltd., Rowville, VIC 3178, Australia

**Keywords:** flesh colour, flesh firmness, index of absorbance difference (I_AD_), machine learning, non-destructive measurements, pigments, sensor, ripeness, skin colour, soluble solids

## Abstract

Technology for rapid, non-invasive and accurate determination of fruit maturity is increasingly sought after in horticultural industries. This study investigated the ability to predict fruit maturity of yellow peach cultivars using a prototype non-destructive fluorescence spectrometer. Collected spectra were analysed to predict flesh firmness (FF), soluble solids concentration (SSC), index of absorbance difference (I_AD_), skin and flesh colour attributes (i.e., a* and H°) and maturity classes (immature, harvest-ready and mature) in four yellow peach cultivars—‘August Flame’, ‘O’Henry’, ‘Redhaven’ and ‘September Sun’. The cultivars provided a diverse range of maturity indices. The fluorescence spectrometer consistently predicted I_AD_ and skin colour in all the cultivars under study with high accuracy (Lin’s concordance correlation coefficient > 0.85), whereas flesh colour’s estimation was always accurate apart from ‘Redhaven’. Except for ‘September Sun’, good prediction of FF and SSC was observed. Fruit maturity classes were reliably predicted with a high likelihood (*F1*-score = 0.85) when samples from the four cultivars were pooled together. Further studies are needed to assess the performance of the fluorescence spectrometer on other fruit crops. Work is underway to develop a handheld version of the fluorescence spectrometer to improve the utility and adoption by fruit growers, packhouses and supply chain managers.

## 1. Introduction

Fruit maturity indices are used to inform harvest logistics and supply chain management decisions for the delivery of fruit with optimal quality to consumers. Soluble solids concentration (SSC), flesh firmness (FF), starch concentration, titratable acidity, skin and flesh colour, fruit size and shape, ethylene production and respiration rate are useful indices used for stone and pome fruit maturity assessment [1,2]. However, until more recent times, the determination of these parameters has been mostly carried out destructively on small sample sizes, leading to time-consuming operations, expensive labour and often subjective data influenced by operators’ skills. Only in recent years, the introduction of spectrometers has led to the increasing adoption of non-destructive devices for food quality estimation (e.g., near-infrared, fluorescence meters, mid-infrared and multispectral/hyperspectral imagery) [3,4,5,6]. Based on the maturity index of interest, one technology can be more reliable than others. The handheld, non-destructive Delta Absorbance (DA)meter was introduced by Ziosi et al. in 2008 [7] to determine the index of absorbance difference between 670 and 720 nm (I_AD_) and has often been used for maturity estimation in stone fruits thereafter [8,9]. The I_AD_ measures chlorophyll a concentration in peach fruit and it has been shown to correlate with ethylene production and respiration rates [7]. Currently, the Australian stone fruit industry recommends the use of FF and I_AD_ for maturity assessment, whilst SSC and fruit size are mostly used as quality parameters. Near-infrared (NIR) spectrometers have been reliably adopted for the estimation of SSC and dry matter in many different fruits—e.g., apple [10,11,12], pear [13,14], kiwifruit [15] and stone fruits [16,17,18,19]—as different wavelengths in the NIR region are very well correlated with the absorbance and reflectance of water and soluble sugars. Prediction of FF in stone fruits via NIR spectrometry is not as reliable as for SSC and dry matter, as this index is influenced by a combination of several physiological and physical factors (e.g., changes in soluble sugars and structural carbohydrates, pectins and physical damage) and does not consistently correlate with specific spectral wavelengths [19]. Other non-destructive technologies such as magnetic resonance, although very precise for the estimation of some maturity indices [20], remain too costly for wide-scale use by industry.

In yellow peaches, skin ground colour and flesh colour have been previously associated with maturity [1,21,22,23,24,25]. Both these parameters are not susceptible to the influence of light. Therefore, during the ripening process, their colour typically turns from green to yellow to orange-red. However, several new yellow peach cultivars have been bred for uniform red skin colouration, that in combination with high SSC represent important key quality parameters for consumers in Australia and south-eastern Asian markets—the most relevant export destinations for Australian stone fruits. This has led to reduced presence or absence of skin ground colour in some cultivars, making skin colour not an ideal candidate for maturity determination in yellow peach cultivars. On the other hand, flesh colour is a more stable attribute among yellow peach cultivars, but its determination requires sample destruction.

Flesh colour is traditionally determined by visual assessments, but colour-measuring devices can be used to determine colour attributes in the CIELAB space [26]. This three-dimensional space is characterised by L* (i.e., a lightness coefficient ranging from 0 (black) to 100 (white)), a* (i.e., a scale of redness to greenness ranging from −60 (true green) to + 60 (true red)) and b* (i.e., a scale of yellowness to blueness ranging from −60 (true blue) to + 60 (true yellow)) [27]. Hue angle (H°) and chroma (C*) colour attributes can be calculated from L*, a* and b* [28]. The H° is calculated as the arc tangent of b*/a* and represents a 360° wheel where 0 or 360° is true red, 90° is true yellow, 180° is true green and 270° is true blue; C* represents colour saturation or “vividness”—diluted with white or darkened with black, with more positive values being brighter and more negative values being duller and is calculated as the square root of (a*)^2^ + (b*)^2^ [29]. Flesh a* has been associated with maturity in clingstone peaches [21,22] and, more specifically, to carotenoid content, the most characteristic pigment in the pulp of yellow peach [24]. However, being on a scale from green to red, a* does not take into consideration the yellow colour component—mostly given by carotenoids—that is instead part of H°. Therefore, flesh H° has also been linked to maturity in yellow peach [30] and apricot [31]. In mature yellow peaches, flesh tends to be more orange or red than immature fruit, with a difference of approximately 5°H [30]. Slaugther et al. [30] also observed that flesh H° is a better indicator of maturity than flesh firmness in yellow clingstone peaches. Skin or flesh H° in yellow peaches was predicted using a Vis/NIR interactance spectrometer [32] and a portable fluorometer [26]. Fluorescence spectroscopy has been linked to the concentration of pigments such as chlorophylls, anthocyanins, flavonols and carotenoids in fruit tissues [33,34,35], with these pigments showing reflectance and absorbance features in the 400–800 nm spectral range [36,37].

The general aim of this study was to assess the ability to non-destructively predict the maturity of yellow peach cultivars using a fluorescence spectrometer. More specifically, the accuracy of the prediction of well-established and less-adopted maturity indices expressed in continuous variables such as SSC, FF, I_AD_, skin and flesh colour attributes, and three maturity classes expressed in a categorical variable (i.e., immature, harvest-ready and mature) were investigated. The hypothesis was that multivariate analyses of pigment fluorescence spectra may lead to accurate estimations of maturity (>75%).

## 2. Materials and Methods

### 2.1. Experimental Site, Plant Material and Fruit Sampling

The experiment was carried out at the Tatura SmartFarm, Agriculture Victoria, Australia (36°26′7″ S and 145°16′8″ E, 113 m a.s.l.) between January and March 2020 on 5–7 years old trees of four yellow peach cultivars (*Prunus persica* L. Batsch, ‘August Flame’, late ‘O’Henry’, ‘Redhaven’ and ‘September Sun’). The experimental orchard’s soil had clay-loam texture and trees were irrigated, fertigated, pruned and pest/disease managed following established local commercial practices. Fruit of the four cultivars were harvested based on the I_AD_ maturity classes reported by the Victorian Horticulture Industry Network [38]. A DA-meter (TR Turoni, Forlì, Italy) was used to determine the I_AD_ and fruit were harvested at the harvest-ready (onset of ethylene) maturity stage [38], with the exception of ‘Redhaven’ fruit that were harvested late (i.e., mature—peak of ethylene emission) due to temporary unavailability of the DA-meter. Two hundred fruit per cultivar were picked in mid-January 2020 (‘Redhaven’), mid-February 2020 (‘O’Henry’ and ‘August Flame’) and early-March 2020 (‘September Sun’). Fruit were hand-picked early in the morning (i.e., before 0800 h, AEST) from different orchard rows and trees, and with a sampling strategy of selecting a wide range of fruit size, shape and skin red colour coverage within each cultivar. After harvest, fruit were brought to the laboratory and left to adjust to a constant temperature of 25 °C for approximately two hours. Meanwhile, one cheek per fruit was marked and specimens were numbered progressively from 1 to 200. All the fruit non-destructive and destructive measurements were carried out within 5–8 h of harvest.

### 2.2. Fluorescence Spectroscopy

Fluorescence spectra were collected on the marked cheek of each fruit using a custom-built fluorescence spectrometer prototype (Rubens Technologies Pty Ltd., Rowville, VIC, Australia) featuring excitation UV LED sources emitting at a wavelength of 400 nm. The spectral sensitivity of the device was in the range 350–950 nm. Since the fluorescence emission is orders of magnitude weaker than the excitation UV intensity, a high-pass filter with cut-off wavelength of 500 nm was placed at the spectrometer entrance to cut the primary UV light and retain only the fluorescent emission from the sample. Recorded spectra represented the average of five readings per fruit obtained in 5–10 s. Data were collected using a custom software running on the Windows 10 operating system and via a USB connection to a laptop.

Spectral measurements were carried out inside a black enclosure to remove influence from external light. The distance between the spectrometer entrance window and the surface of the fruit was kept constant at a value of 80 mm. The measured spectra were normalised to the total emission.

### 2.3. Determination of Maturity Indices and Maturity Classes

Fruit equatorial diameter from cheek to cheek (FD, mm) and fruit weight (FW, g) were obtained using a digital calliper and a digital scale with two decimal places, respectively. Subsequently, the marked cheek of each individual fruit previously exposed to the fluorescence spectrometer was scanned with the DA-meter to obtain the I_AD_ and data were downloaded into a PC using an internal micro-SD card. Fruit were classified into three maturity classes based on cultivar-specific I_AD_ thresholds obtained from relationships with fruit ethylene emission [38], as shown in Table 1.

Skin colour was determined on a single point per fruit—in the centre of the marked cheek—using a portable spectrophotometer (Nix Pro^TM^, Nix Sensor Ltd., Hamilton, ON, Canada) with a 14 mm aperture, a D50 illuminant and a 2° observer angle. The Nix Pro^TM^ spectrophotometer was operated using the Nix Pro^TM^ Color Sensor application on an Android smartphone via Bluetooth connectivity. Data were logged in the smartphone memory and downloaded at the end of each measurement session. Colour data was stored in the RGB, XYZ, CMYK, HEX and CIELAB formats, but only the latter was used in this study (i.e., L*, a*, b*, C* and H°). Next, fruit skin was removed from the marked cheek using a potato peeler and the flesh was immediately scanned for colour assessment using the Nix Pro^TM^ spectrophotometer with the same methodology adopted for skin colour determination.

The portion of fruit flesh that was scanned with the Nix Pro^TM^ was then exposed to a penetrometer (FT327, FACCHINI srl, Alfonsine, Italy) with an 8 mm tip and FF was measured on a scale from 0 to 15 kgf. Lastly, a few drops of juice (~2 mL) were extracted from the same area and measured with a digital refractometer (PR-1; ATAGO CO., LTD., Saitama, Japan) to obtain SSC (°Brix).

### 2.4. Data Analysis and Modelling

#### 2.4.1. Maturity Statistics

FD, FW, FF, SSC, I_AD_, skin and flesh colour attributes (i.e., L*, a*, b*, C* and H°) were compared among the four cultivars using one-way analysis of variance (ANOVA), followed by the Games–Howell post hoc test for comparison of groups with unequal variances.

The correlations between pairs of the FD, FW, FF, SSC, I_AD_, skin and flesh colour attributes was tested using Pearson’s correlation coefficients (*r*) and presented in a correlation heatmap. From this point onwards, a* and H° were the only colour attributes considered for further data analyses, as b*, C* and L* are less important for yellow peach fruit characteristics.

Linear regression models of flesh a* and H° against I_AD_ were calculated to verify the validity of these two flesh parameters for maturity assessment. In addition, these models were used to estimate a* and H° maturity thresholds corresponding to I_AD_ maturity classes in the four peach cultivars (Table 1) in order to provide a clear range of a* and H° values for harvest-ready fruit, as these two parameters are not established in the stone fruit industry. ANOVA, post hoc tests and Pearson’s correlations were carried out using R (v. 4.0.2) [39] and the “Userfriendliscience” package [40]. Graphs were generated using SigmaPlot 12.5 (Systat software Inc., Chicago, IL, USA).

#### 2.4.2. Prediction Modelling

To achieve maturity prediction this work focused on analysing the fluorescence spectra using two different machine learning approaches. The first approach was used for the prediction of continuous variables such as FF, SSC, I_AD_, skin and flesh a* and H°. The second approach was put in place to predict fruit maturity classes (as shown in Table 1) that represent simple categories that are widely understood by growers and industry stakeholders.

Prediction models for FF, SSC, I_AD_ and the most relevant colour attributes in both skin and flesh (a* and H°) were carried out using partial least square (PLS) regression with spectral band optimisation. Fluorescence spectra were not used for the prediction of FD and FW, as no relationship between fluorescence and fruit size was expected. All models were optimised using a k-fold cross-validation procedure using k = 10 splits. The basic idea was to select the most informative wavelength bands out of the entire spectral response. The selection was done via a random optimisation procedure called simulated annealing (SA) [41]. This algorithm begins with a randomly selected set of bands and, at each iteration, randomly changes a subset of these bands. A PLS regression model was developed at each iteration and the Akaike information criterion (AIC) [42] was used as the cost function. The aim of the algorithm was to decrease the cost functions, hence, to improve the model. The coefficients of determination for the cross-validation procedure (*R*^2^_CV_) and Lin’s concordance correlation coefficients (*r*_c_) [43] were calculated to assess models’ robustness, with the latter being a reliable measure of the agreement between two variables. The *r*_c_ is particularly useful when comparing two measures of the same variable, such as when *x* (observed values) and *y* (predicted values) are in the same unit. The best models had the highest agreement between *x* and *y* and generated a *r*_c_ closer to 1 in a 0–1 range. The root mean square error in cross-validation (*RMSE*_CV_) was also calculated to provide a measure of error in the same unit of the measured variable.

The SA optimisation procedure is a variant of a greedy optimisation strategy whereby the algorithm seeks to decrease the cost function, but not monotonically. SA allows for the occasional increase of the cost function to reduce the likelihood of the process getting stuck in a local minimum of the parameter space. In order for the SA process to be effective, the increase of the cost function must be occasional; in other words, it must happen with a small probability, governed by a hyper-parameter, which we chose to be equal to 0.1% of the current value of the AIC at each iteration.

The algorithm workflow was as follows:

1. Raw data were smoothed using convolution with a Gaussian kernel with σ = 1.5 units (corresponding to a physical sigma of approximately 3 nm at the centre of the spectrum).
2. Spectra were “re-binned” into 72 wavelength bands obtained by adding 4 contiguous wavelength bins.
3. The resulting spectra were then mean-centred and scaled to unit variance.
4. After these pre-processing steps the spectra were fed in the SA algorithm. The algorithm starts with a random draw of 20 bands (out of the total 72). At each iteration, a subset of two bands were randomly swapped, a PLS model was developed and cross-validated on the collection of bands selected by the SA. The optimum PLS model at each step was obtained by minimising the *AIC*. The algorithm was run for 5000 iterations.

The number of selected bands (20 in our case) was fixed for all models and initially chosen in accordance with the empirical optimal value of the latent variables selected by the cross-validation process. As such value was of the order of 10, we chose the number of bands to be double that, to provide some redundancy for the dimensionality reduction of the PLS algorithm. In other words, if the number of bands was to be further decreased, the number of latent variables tended to become similar to the number of bands, hence the model became over-constrained. *R*^2^_CV_, *r*_c_, *RMSE*_CV_, AIC and number of latent variables (LV) in the models were presented for the preferred PLS models.

Lastly, the selected 20 wavelength bands were also used to predict the fruit maturity classes (Table 1) in fruit of the four peach cultivars pooled together (*n* = 800) using a linear discriminant analysis (LDA). A three-class confusion matrix was used to visualise the performance of the model. The overall model’s accuracy was based on the likelihood to predict the correct maturity class and expressed in *F1*-scores (0–1 range, where 1 is perfect prediction and 0 is no correct predictions) for each maturity class and for the whole model. The *F1*-score was calculated as the harmonic mean of precision and recall. Precision represents the number of true positive results divided by the sum of true and false positive results. Recall represents the number of true positive results divided by the sum of true positive results and false negative results. The model’s error was calculated as 1 − *F1*.

The algorithms were implemented in Python^TM^ 3.7.6 using the PLS regression and LDA routines available in the Scikit-learn package (v. 0.22.2) [44]. Sample scripts of the algorithms are freely available as a Project Jupyter Notebook [45].

## 3. Results

### 3.1. Fruit Maturity Indices

#### 3.1.1. Comparisons between Cultivars

Maturity indices were found to be significantly different between the four yellow peach cultivars (Table 2). ‘September Sun’ had the largest FD and FW, whereas ‘Redhaven’ fruit were the smallest. Since ‘Redhaven’ fruit were harvested late, when already mature, their FF and I_AD_ were significantly lower than in other cultivars (Table 2). In ‘August Flame’, ‘O’Henry’ and ‘September Sun’, the median I_AD_ was within the respective harvest-ready I_AD_ range reported in Table 1. ‘O’Henry’ fruit had the highest sugars (median SSC of 14.8°Brix), whilst ‘Redhaven’ fruit were the least sweet (11.9°Brix).

Skin colour was significantly different between the cultivars under study (Table 2). ‘Redhaven’ fruit had the significantly highest skin L* (i.e., lighter colouration), a* (i.e., redder than other cultivars in the green-red scale), b* (i.e., more yellow than other cultivars in the blue-yellow scale), C* (i.e., higher colour vividness than other cultivars) and H° (i.e., more orange-red than other cultivars in the red to yellow scale, i.e., 0 to 90°).

Flesh colour was also found to be significantly different between ‘August Flame’, ‘O’Henry’, ‘Redhaven’ and ‘September Sun’ (Table 2). Even in this case, the flesh of ‘Redhaven’ fruit had the highest L*, a*, b* and C*, but the lowest H° when compared to the other cultivars.

#### 3.1.2. Correlations between Maturity Indices

**Figure 1:**
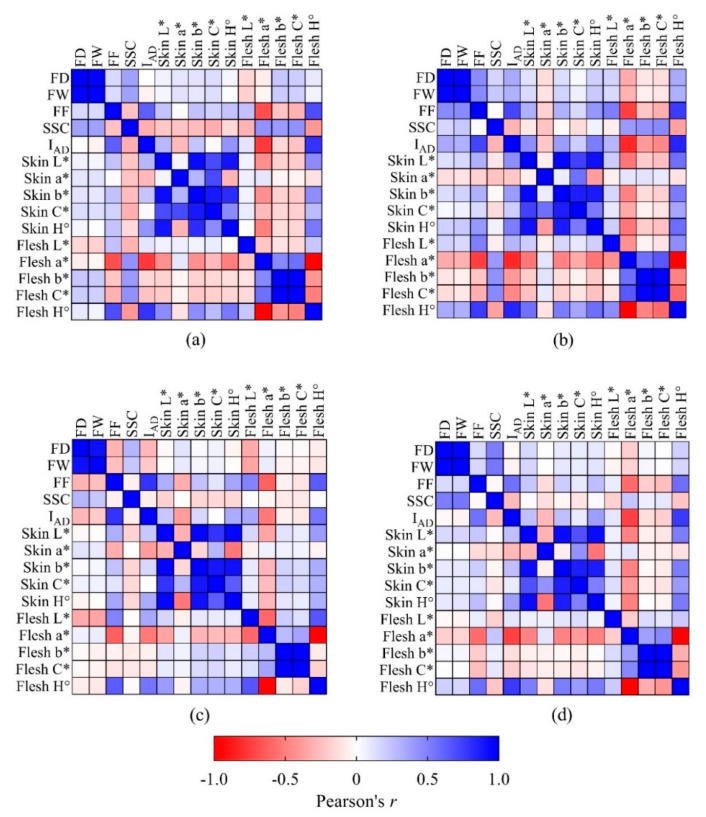
Pearson’s correlation (*r*) heatmap for FD, FW, FF, SSC, I_AD_ and skin and flesh colour attributes (L*, a*, b*, C*, H°) in fruit of four yellow peach cultivars at harvest (*n* = 200). Correlation strength represented on a colour scale, where red represents high negative correlation, blue corresponds to high positive correlation and white is no correlation. (**a**) ‘August Flame’; (**b**) ‘O’Henry’; (**c**) ‘Redhaven’ and (**d**) ‘September Sun’.

Figure 1 highlights a colour-coded correlation strength between pairs of parameters, with darkest colours associated to strong correlations (blue for *r* ≥ 0.75 and red for *r* ≤ −0.75, *p* < 0.001) and white colour representing absent correlation (−0.10 < *r* < 0.10, *p* > 0.05). A strong correlation was observed between FW and FD in all the cultivars (Figure 1), as these two fruit size parameters are logically directly associated. In addition, strong correlation within skin colour attributes or within flesh colour attributes were not considered, as they are a consequence of collinearity among variables that are derived from their interdependency due to the fact that some of these variables are calculated from others (e.g., when a* increases, H° decreases, when C* increases, b* increases). SSC was not strongly correlated with other parameters (*r* < 0.50 or >−0.50), whereas FF and I_AD_ had an association with *r* > 0.50 in all the cultivars. FF and I_AD_ were in turn highly correlated (*r* > 0.50) with the flesh colour attributes a* and H°. Flesh a* was negatively correlated with I_AD_, whereas flesh H° had a positive relationship.

Although ‘Redhaven’ fruit were picked at a late maturity stage, correlations between parameters had in most cases similar strengths to those observed in ‘August Flame’, ‘O’Henry’ and ‘September Sun’ (Figure 1).

**Figure 2:**
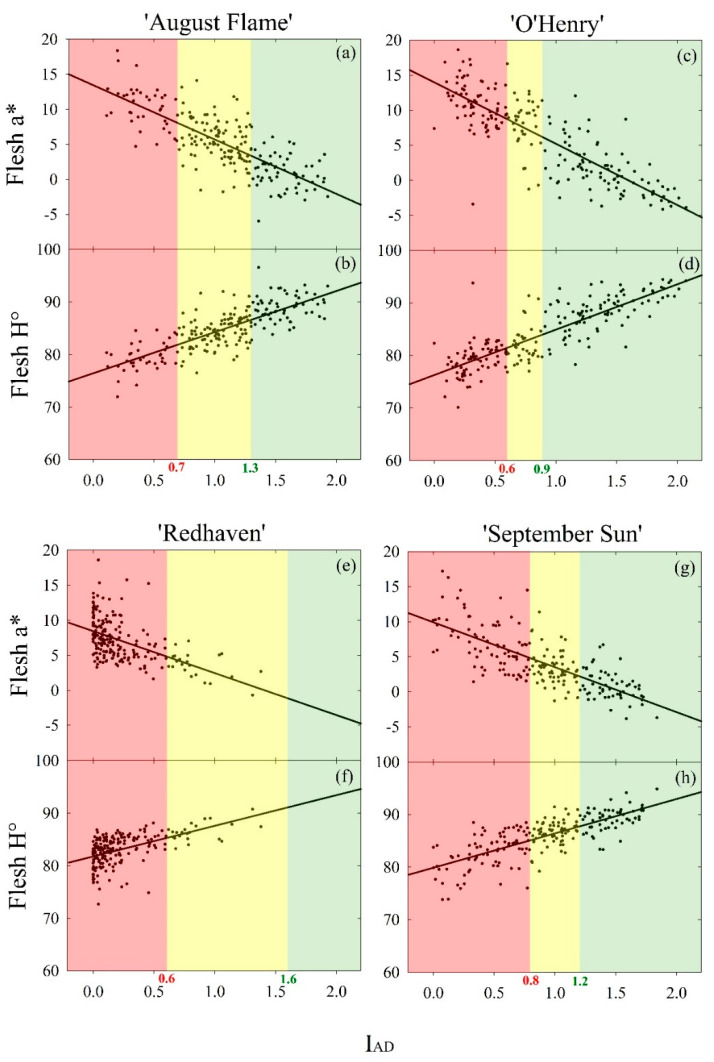
Scatter plots and linear regression fits of flesh a* and H° against the I_AD_ in fruit (*n* = 200) of four yellow peach cultivars at harvest (see Table 3 for regression coefficients). The three colours indicate I_AD_ maturity classes reported in Table 1: (green) immature—no ethylene emission; (yellow) harvest-ready—onset of ethylene emission and (red) mature—ethylene emission peak. (**a**) Flesh a* and (**b**) H° vs. I_AD_ in ‘August Flame’; (**c**) Flesh a* and (**d**) H° vs. I_AD_ in ‘O’Henry’; (**e**) Flesh a* and (**f**) H° vs. I_AD_ in ‘Redhaven’ and (**g**) Flesh a* and (**h**) H° vs. I_AD_ in ‘September Sun’.

The scatter plots with linear fits in Figure 2 highlight the relationships of flesh a* and H° with I_AD_ in the four yellow peach cultivars. Table 3 shows linear regression models in which the I_AD_ maturity threshold values reported in Table 1 were set as *x* to predict a* and H° thresholds between maturity classes in the four cultivars under study. ’Redhaven’s’ linear regression model generated relatively low *R*^2^ (<0.50) for both a* and H°, as expected from correlation heatmaps (Figure 1), whereas in the other cultivars the models’ *R*^2^ were higher than 0.50. The range of a* and H° values in harvest-ready fruit was relatively small in each of the four cultivars—from 2.6 (‘September Sun’) to 6.0 (‘Redhaven’) for a* and from 2.6 (‘September Sun’) to 5.8 (‘Redhaven’) for H°. Maturity thresholds appeared to be cultivar-specific, although ‘August Flame’ and ‘O’Henry’ fruit had almost identical a* and H° thresholds. The linear regression slopes were similar but inverse for a* and H°.

### 3.2. Fluorescence Spectra and Maturity Prediction

#### 3.2.1. Spectra Characteristics

**Figure 3:**
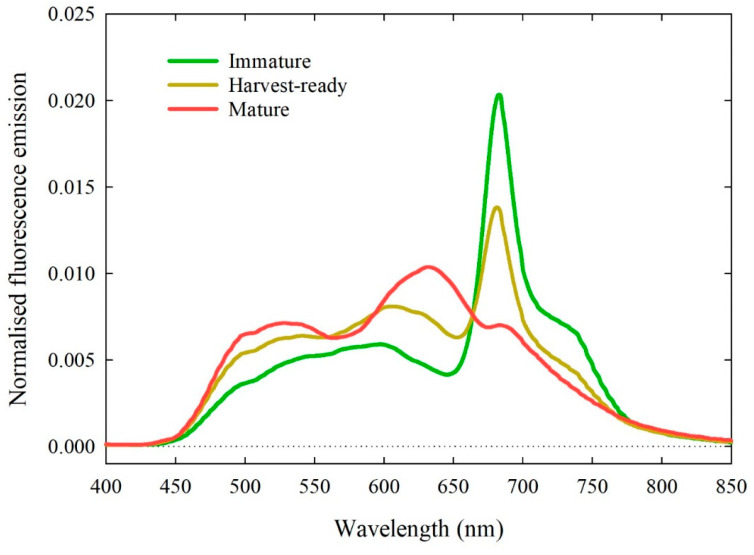
Normalised fluorescence emission spectra for three individual ‘August Flame’ fruit at different maturity classes. Immature: Index of absorbance difference (I_AD_) > 1.3; harvest-ready: 1.3 ≤ I_AD_ ≤ 0.7 and mature = I_AD_ < 0.7 (see Table 1).

To highlight the response of fluorescence emission under different maturity classes (based on the classification in Table 2) Figure 3 shows spectra of three individual ‘August Flame’ fruits. The immature and harvest-ready fruit emitted fluorescence peaks at ~680–690 nm, with the latter being sensibly lower than the former. However, the harvest-ready fruit showed higher emission between 450 and 650 nm compared to the immature fruit. The mature fruit had no clearly defined peaks, but instead showed a hump with maximum emission between 600 and 650 nm, a smaller but wider hump in the 480–550 nm range and a barely visible hump at 680–690 nm. Both the humps at 600–650 and 480–550 nm observed in the mature fruit were more pronounced than in the harvest-ready fruit.

Measured maturity indices corresponding to the fruit shown in Figure 3 are reported in Table 4. The immature fruit had the highest FF, SSC, I_AD_, skin and flesh H°, and the lowest skin a* and flesh a*. This trend was expectedly inverted in mature fruit, in line with previous results (Figure 1).

#### 3.2.2. PLS Models

**Figure 4:**
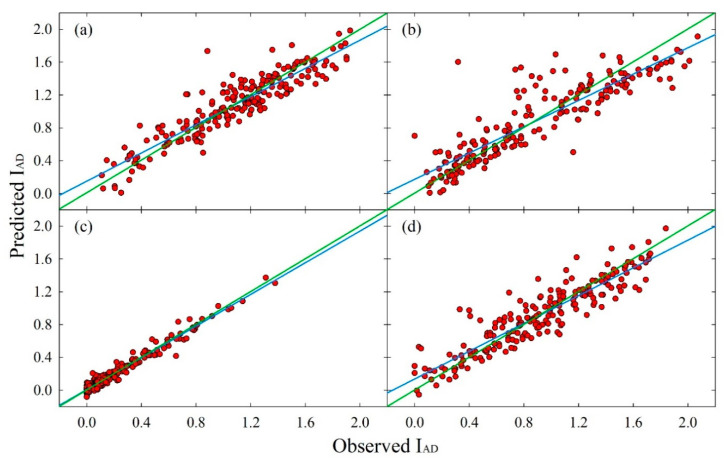
Scatter plots of cross-validation predicted I_AD_ against observed I_AD_ in yellow peach fruit (*n* = 200) using a partial least square regression (PLS) model: (**a**) ‘August Flame’; (**b**) ‘O’Henry’; (**c**) ‘Redhaven’ and (**d**) ‘September Sun’. Blue lines represent partial least square regression model linear fits; green lines represent reference linear fits where predicted I_AD_ = observed I_AD_. PLS statistics are reported in Table 5.

Once fluorescence emission spectra were processed with the PLS algorithm, predicted values of FF, SSC, I_AD_, skin a* and H° and flesh a* and H° were obtained and compared to measured data. Figure 4 shows cross-validation predicted I_AD_ plotted against the observed I_AD_. A visual assessment of the concordance between the two variables suggested that the PLS models provided a good estimation of I_AD_, as model linear fits were relatively close to a *y* = *x* reference line. Linear fits also show that the PLS models in the four cultivars slightly overestimated I_AD_ when its values were low—i.e., mature fruit—and underestimated them when they were high—i.e., immature fruit. However, overestimations at a minimum observed I_AD_ = 0.0 (+0.17, +0.15, +0.14 and 0.01 in ‘O’Henry’, ‘August Flame’, ‘September Sun’ and ‘Redhaven’, respectively) and underestimations at a maximum observed I_AD_ = 2.2 (−0.27, −0.20, −0.16 and −0.07 in ‘O’Henry’, ‘September Sun’, ‘August Flame’ and ‘Redhaven’, respectively) were considered very low. The model’s linear fit in ‘Redhaven’ fruit was the closest to the *y* = *x* line, although most of the points were found at low I_AD_.

The calculated *R*^2^_CV_, *r*_c_ and *RMSE*_CV_ provided valuable information to better assess prediction power and error of the PLS models. A summary of these parameters is presented in Table 5 and highlight differences in models’ robustness among cultivars. AIC values cannot be compared across cultivars and maturity indices, as they are only meant to be used to compare alternative models for the same dataset. The PLS model for FF and SSC predictions generated the overall lowest *R*^2^_CV_ and *r*_c_ among maturity indices. The fluorescence emission spectra could only poorly predict SSC and FF in ‘September Sun’ fruit (*R*^2^_CV_ ≤ 0.50 and *r*_c_ < 0.70). I_AD_ was the only maturity index that was consistently and strongly predicted by the PLS models in all the cultivars (*R*^2^_CV_ ≥ 0.80 and *r*_c_ ≥ 0.89), regardless of maturity advancement, with *RMSE*_CV_ < 0.25. *R*^2^_CV_ and *r*_c_ were also consistently in the 0.75–0.88 and 0.85–0.94 ranges, respectively, in models for the prediction of skin a* and H°, with the latter being always the one with highest precision between the two-colour attributes. Conversely, the accuracy of flesh a* and H° prediction differed in the four cultivars, ranging from the most precise predictions in ‘O’Henry’ (*R*^2^_CV_ = 0.87 and *r*_c_ = 0.93) to the poorest estimates in ‘Redhaven’ (*R*^2^_CV_ < 0.50 and *r*_c_ ≤ 0.65).

#### 3.2.3. LDA Model

**Figure 5:**
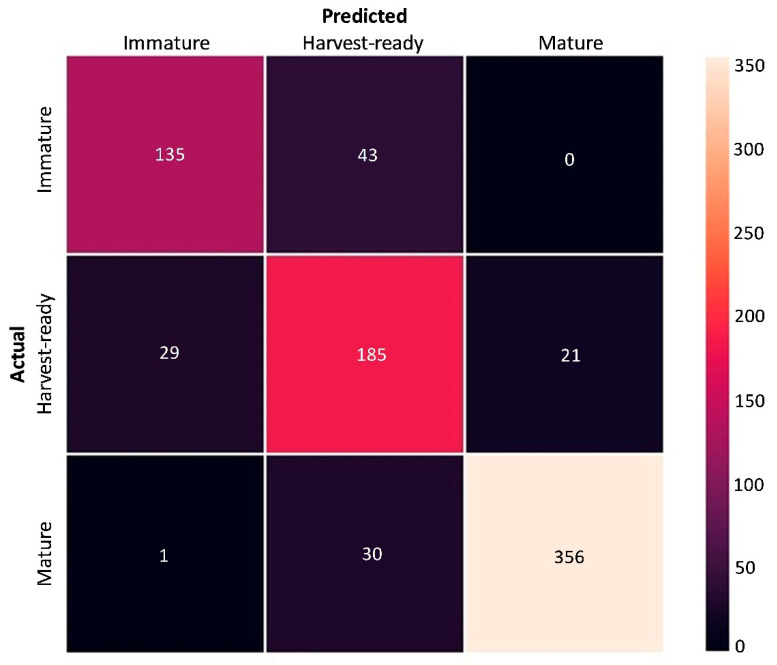
Confusion matrix of actual and predicted maturity classes generated after a linear discriminant analysis of the fluorescence spectra obtained from fruit of the four peach cultivars ‘August Flame’, ‘O’Henry’, ‘Redhaven’ and ‘September Sun’ pooled together (*n* = 800). Numbers in each cell represent fruit counts for a specific predicted-actual combination. Numbers in the diagonal top-left to bottom-right axis represent true positives of the prediction.

The LDA model for the pooled fruit dataset generated an overall *F1* = 0.85; thus, the overall likelihood to estimate the correct maturity class was of approximately 85%, with an estimate error of 15%. The confusion matrix in Figure 5 shows that the model estimated 356, 185 and 135 true positives in mature, harvest-ready and immature fruit, when actual counts for the three classes were 387, 235 and 178, respectively. The single-class *F1*-scores were 0.93, 0.79 and 0.75 for mature, immature and harvest-ready fruit, respectively. The two opposite diagonal values in the black matrix cells show that only one mature fruit was classified as immature, and no immature fruit were classified as mature, providing additional strength to the model’s results.

## 4. Discussion

The four cultivars selected for this study produced fruit with different characteristics, as highlighted in Table 2. The skin colour values reported for ‘Redhaven’ were partially influenced by advanced maturity due to the late sampling of fruit at harvest. However, skin colour was measured on the fruit cheek along the equatorial diameter, usually dominated by cover colour rather than ground colour, with the former being poorly influenced by maturity. Therefore, significantly different skin colouration among cultivars was most likely influenced by intrinsic genotypic characteristics. In flesh colour measurements, lower H° represented redder flesh, as also suggested by a* (Table 2), a typical consequence of fruit maturation. Therefore, flesh colouration differences in the four cultivars may be attributed to both genotypic characteristics and maturity stage.

The fact that SSC was not strongly correlated with other parameters (Figure 1) suggests that sugars are a relatively independent fruit quality parameter that should be used with caution when attempting to assess maturity in yellow peaches. The strong correlations between FF and I_AD_ indicate that there is a degree of interdependency between loss of firmness and chlorophyll degradation in yellow peaches. In addition, the strong correlation of chlorophyll degradation with the flesh colour attributes a* and H° was probably governed by the changes in flesh pigments, with an overall decrease of chlorophyll over the maturation period. An exception was highlighted in ‘Redhaven’, where the correlations between I_AD_ and flesh a* and H° were poorer than in other cultivars, probably influenced by the fact that when fruit are more mature, I_AD_ saturates towards its minimum value (i.e., 0.0) and fails to detect further physiological changes (e.g., cell wall and pectin modifications, internal darkening) that might instead be captured by variations in flesh colour attributes such as a* and H°. Our results support earlier work by Slaughter et al. [30], who observed a difference of approximately 5°H of flesh hue between immature and mature peaches. The linear models shown in Table 3 highlight that the flesh colour attributes a* and H° may be used as maturity indices, although thresholds between maturity classes are cultivar specific. The similar but inverse slope in their relationship with I_AD_ suggests that regression models based on one flesh colour attribute or the other are likely to have similar accuracy. This explains why literature shows the use of both a* and H° for fruit maturity assessments [30,46].

Typically, in immature fruit most of the fluorescence is emitted by chlorophyll pigments. Chlorophyll fluorescence has a primary peak at ~683 nm and a secondary peak at ~720 nm. The width of the peaks in stone fruit is usually comparable to their separation, so that the secondary peak appears as a shoulder between 700 and 750 nm [47] These features are clearly visible in immature and harvest-ready ‘August Flame’ fruit (Figure 3). In mature fruit, since chlorophyll had mostly degraded, its fluorescence emission is greatly diminished, and the measurable signal at ~683 nm is lower than the maximum emission emitted between 600 and 650 nm. The fluorescence emission observed at 600–650 nm is likely to be due to anthocyanins in the skin, as this range is compatible with fluorescence emission ranges of the 3-glucoside of malvidin (i.e., oenin) [48], an anthocyanin found in red grapes. Fluorescence emission at wavelength < 600 nm can be explained by a combination of flavonols and carotenoids. Flavonols such as quercetin, mostly found in yellow peach peels and scarcely present in flesh [49,50], emit green fluorescence with maxima near 520–530 nm [51]. Carotenoids have maximum fluorescence emission in the 450–550 nm range [52] and they are mainly present in the form of *β*-carotene and *β*-cryptoxanthin in yellow peach flesh [53]. On the one hand, the immature fruit showing a large chlorophyll peak at ~683 nm had, expectedly, the lowest skin and flesh a* and the highest skin and flesh H°, i.e., greener (Table 4). On the other hand, the mature fruit showed the highest emission in the flavonol and carotenoid spectral ranges (Figure 3), meaning that these pigments were the most likely candidates to induce high a* and low H° in both skin and flesh (i.e., redder) (Table 4). Changes of pigments concentrations in skin and flesh over fruit maturation are the basis for maturity prediction by fluorescence spectrometry.

PLS models highlighted that fluorescence emission can estimate FF and SSC in yellow peaches at harvest, although prediction is not very accurate (Table 5). I_AD_ and skin a* and H° were consistently predicted in all the cultivars with good accuracy (*R*^2^_CV_ ≥ 0.75 and *r*_c_ ≥ 0.85). The prediction power for flesh a* and H° diverged among cultivars, with models for ‘Redhaven’—the most mature fruit—showing low accuracy (*R*^2^_CV_ < 0.50 and *r*_c_ ≤ 0.65, Table 5). The high median values of skin L* and C* in ‘Redhaven’ fruit (Table 2) may have led to increased self-absorption of the fluorescence from the skin, hence, a decreased contribution of the pulp emission to the total measured fluorescence. This in turn, could have caused a reduction of the prediction ability of fluorescence spectrometry for flesh a* and H° in this cultivar.

The LDA model’s *F1*-score was equal to 0.85, which represented a very good likelihood of predicting the correct maturity class. All the class predictions showed robust predictions (*F1* ≥ 0.75). The lowest *F1* (0.75) was observed in the intermediate maturity class (harvest-ready) as in this case there were more chances to have a false positive on both sides of the class, i.e., neighbouring mature and immature classes. The highest accuracy obtained from the prediction of mature fruit (*F1* = 0.93) was likely to be influenced by the larger sample size for this class (*n* = 387) compared to the other classes. The PLS models obtained for I_AD_ and the LDA models obtained for maturity classes satisfied the hypothesis of our study, as they generated maturity predictions with accuracies ≥ 0.85, in terms of *r*_c_ and *F1*-score, respectively.

The fluorescence spectrometer used in this study captures the steady-state fluorescence from the fruit under constant excitation light illumination. This system is fundamentally different from the pulse-amplitude modulation (PAM) fluorometers [54], which employ modulated excitation and fast detection to decouple fluorescence yield information from the measured fluorescence intensity. The sensitivity of a PAM fluorometer is derived from its time resolution and it is usually restricted to chlorophyll fluorescence. The fluorescence spectrometer described here does not produce fluorescence yield information, but it enables rapid measurements of a large spectral band without requiring specific sample preparation, most notably without requiring dark adaption of the fruit samples.

This characteristic is especially promising in view of field applications using hand-held devices, as the spectra can be captured without picking the fruit. The development of a wireless, battery-operated, handheld device for field estimation of fruit maturity is presently underway and early prototypes are being tested in field pilots of stone fruits (nectarine, white peach, plum, apricot) and pome fruits (apple, pear). While the current system gives good results in maturity estimation, the prediction of commercial maturity parameters such as SSC or FF is somewhat limited and very dependent on the cultivar. This suggests that a combination of fluorescence and NIR is required for improved performances. At the same time, a better modelling of the self-absorption properties of the fruit skin may help compensate from the signal reduction from the pulp, hence improve the model accuracy.

The ability to correctly predict maturity classes, though amenable to improvements, is suggestive of a post-harvest application of the fluorescence principle in graders. This application is currently being explored, in order to take advantage of prediction models that have the potential to be, to a large extent, independent from cultivar information within the same crop (e.g., yellow peach), hence better suited to a commercial operation with constantly changing cultivars being processed over a season.

## 5. Conclusions

In conclusion, the fluorescence spectrometer used in this study accurately predicted fruit maturity in yellow peaches. The machine learning prediction algorithms have the potential to be easily implemented in different configurations of the spectrometer (e.g., fluorescence alone, fluorescence + NIR, fluorescence + RGB and NIR in grading machines, portable or benchtop) for both in situ and post-harvest fruit maturity estimations. The sensor’s applicability for fruit maturity prediction and for the assessment of fruit quality parameters and storage disorders in stone fruits and apples is currently under investigation at the Tatura SmartFarm.

## Figures and Tables

**Table 1:** Fruit maturity classes for yellow-flesh peach based on cultivar-specific thresholds of index of absorbance difference (I_AD_) according to the Victorian Horticulture Industry Network [38].

Cultivar	Maturity Classes (I_AD_)		
	Immature ^1^	Harvest-Ready ^2^	Mature ^3^
‘August Flame’	I_AD_ > 1.3	1.3 ≤ I_AD_ ≤ 0.7	I_AD_ < 0.7
‘O’Henry’	I_AD_ > 1.2	1.2 ≤ I_AD_ ≤ 0.7	I_AD_ < 0.7
‘Redhaven’	I_AD_ > 1.6	1.6 ≤ I_AD_ ≤ 0.6	I_AD_ < 0.6
‘September Sun’	I_AD_ > 1.2	1.2 ≤ I_AD_ ≤ 0.8	I_AD_ < 0.8

^1^ undetectable ethylene emission, ^2^ onset of ethylene emission and ^3^ ethylene emission peak.

**Table 2:** Fruit diameter (FD), fruit weight (FW), flesh firmness (FF), soluble solids concentration (SSC), I_AD_ and skin and flesh colour attributes (L*, a*, b*, C*, H°) in fruit of four yellow peach cultivars at harvest (*n* = 200). Medians and standard deviations (in brackets) are presented. Different letters represent significant differences (*p* < 0.01) between cultivars based on analysis of variance (ANOVA) followed by the Games–Howell post hoc test.

Maturity Index/Colour Attribute	Cultivar			
	‘August Flame’	‘O’Henry’	‘Redhaven’	‘September Sun’
FD (mm)	62.4 (5.5) c	64.9 (4.6) b	52.8 (5.4) d	70.2 (7.3) a
FW (g)	127.7 (30.4) c	146.7 (26.3) b	77.6 (24) d	186.3 (54.2) a
FF (kgf)	7.2 (1.8) a	6.2 (2.1) b	1.9 (1.5) c	6.1 (1.7) b
SSC (°Brix)	13.2 (2.3) b	14.8 (2.3) a	11.9 (1.3) c	13.4 (2.2) b
I_AD_	1.1 (0.4) a	0.8 (0.5) b	0.1 (0.3) c	0.9 (0.4) b
Skin L*	48.0 (6.4) c	51.9 (7.1) b	56.2 (10.7) a	52.0 (8.3) b
Skin a*	27.5 (6.5) b	28.1 (6.4) b	30.6 (7.7) a	29.8 (6.7) ab
Skin b*	25.8 (8.3) b	25.9 (9.0) b	30.8 (11.6) a	29.9 (9.1) a
Skin C*	39.1 (8.5) c	40.2 (8.2) c	47.6 (9.3) a	43.5 (7.8) b
Skin H°	40.1 (9.7) b	39.4 (11.0) ab	42.4 (13.7) a	42.4 (11.4) ab
Flesh L*	78.5 (2.8) b	78.4 (3.1) bc	81.3 (6.5) a	77.8 (2.7) c
Flesh a*	4.9 (4.3) bc	7.4 (5.6) ab	6.7 (3.1) a	3.2 (3.8) c
Flesh b*	55.1 (3.5) c	55.9 (4.2) b	58.3 (4.2) a	54.8 (3.2) c
Flesh C*	55.6 (3.7) c	56.4 (4.5) b	58.7 (4.2) a	54.9 (3.4) c
Flesh H°	84.9 (4.3) ab	82.7 (5.5) bc	83.3 (2.9) c	86.5 (3.8) a

**Table 3:** Linear regression models and predicted thresholds (*y*) of flesh a* and H° for the maturity classes “harvest-ready” and “mature” based on the indices of absorbance difference (*x*) reported in Table 1. Data reported for fruit of four yellow peach cultivars at harvest (*n* = 200). Intercept, slope and coefficients of determination (*R*^2^) reported for each linear regression model. Standard errors of intercept and slopes are in brackets.

Cultivar	Flesh Colour Attribute	Linear Regression Models			Predicted Maturity Thresholds	
		Intercept	Slope	*R* ^2^	Harvest-Ready	Mature
‘August Flame’	a*	13.4 (0.5)	−7.8 (0.4)	0.60	3.4	8.0
	H°	76.4 (0.5)	7.8 (0.4)	0.62	86.6	81.9
‘O’Henry’	a*	14.0 (0.4)	−8.8 (0.4)	0.70	3.5	7.8
	H°	76.3 (0.4)	8.6 (0.4)	0.71	86.7	82.3
‘Redhaven’	a*	8.4 (0.2)	−6.0 (0.7)	0.28	−1.1	4.9
	H°	81.8 (0.2)	5.8 (0.6)	0.30	91.1	85.3
‘September Sun’	a*	9.9 (0.4)	−6.4 (0.4)	0.55	2.2	4.8
	H°	79.9 (0.4)	6.5 (0.4)	0.57	87.7	85.1

**Table 4:** FF, SSC, I_AD_, skin and flesh a* and H° in three individual ‘August Flame’ fruit shown in Figure 3.

Maturity Index/Colour Attribute	Immature	Harvest-Ready	Mature
FF ^1^ (kgf)	8.8	6.4	5.1
SSC ^2^ (°Brix)	12.7	12.2	12.4
I_AD_^3^	1.5	1.0	0.4
Skin a*	25.7	32.3	35.2
Skin H°	53.7	37.3	31.6
Flesh a*	− 2.5	5.0	8.9
Flesh H°	92.7	84.7	79.5

^1^ Flesh firmness, ^2^ Soluble Solids Concentration, ^3^ Index of absorbance difference.

**Table 5:** Partial least square regression models for the prediction of FF, SSC, I_AD_, skin and flesh a* and H° in four yellow peach cultivars (*n* = 200) using a custom-built fluorescence spectrometer.

Cultivar	Maturity index	*R* ^2^_CV_^1^	*r*_c_^2^	*AIC* ^3^	*RMSE*_CV_^4^	LV ^5^
‘August Flame’	FF ^1^	0.69	0.82	184	1.48	12
	SSC ^2^	0.72	0.84	301	1.9	12
	I_AD_^3^	0.83	0.91	−655	0.18	10
	Skin a*	0.76	0.86	509	3.21	12
	Skin H°	0.77	0.87	657	4.65	11
	Flesh a*	0.76	0.87	337	2.09	12
	Flesh H°	0.80	0.89	305	1.93	12
‘O’Henry’	FF	0.69	0.81	149	1.15	12
	SSC	0.75	0.86	196	1.55	11
	I_AD_	0.80	0.89	−528	0.24	9
	Skin a*	0.75	0.85	467	3.21	7
	Skin H°	0.83	0.91	629	4.45	9
	Flesh a*	0.87	0.93	317	2.04	10
	Flesh H°	0.87	0.93	296	1.93	9
‘Redhaven’	FF	0.78	0.87	−20	0.71	10
	SSC	0.58	0.74	220	1.34	7
	I_AD_	0.96	0.98	−1126	0.05	9
	Skin a*	0.76	0.87	570	3.75	7
	Skin H°	0.88	0.94	668	4.78	10
	Flesh a*	0.46	0.62	375	2.26	9
	Flesh H°	0.49	0.65	340	2.07	10
‘September Sun’	FF	0.50	0.67	280	1.95	6
	SSC	0.49	0.67	382	2.01	8
	I_AD_	0.83	0.91	−651	0.18	8
	Skin a*	0.76	0.86	508	3.28	7
	Skin H°	0.79	0.88	692	5.21	10
	Flesh a*	0.73	0.84	312	2.01	10
	Flesh H°	0.74	0.85	303	1.97	7

^1^ Coefficient of determination of the cross-validation, ^2^ Lin’s concordance correlation coefficient, ^3^ Akaike information criterion, ^4^ root mean square error of the cross-validation and ^5^ number of latent variables.
